# Listeria Bacteremia Presenting With Cerebral Abscess and Endocarditis in an Elderly Patient With Chronic Immune Thrombocytopenia

**DOI:** 10.7759/cureus.16601

**Published:** 2021-07-24

**Authors:** Olushola O Ogunleye, Vanessa Karimi, Nili Gujadhur

**Affiliations:** 1 Department of Internal Medicine, Vassar Brothers Medical Center, Poughkeepsie, USA

**Keywords:** listeria, endocarditis, brain abscess, bacteremia, chronic immune thrombocytopenia

## Abstract

Central nervous system involvement by *Listeria monocytogenes* usually presents as meningitis, meningoencephalitis or, less frequently, rhombencephalitis. Listerial brain abscesses are rare. Moreover, only 5-8% of listerial bacteremia is complicated by infective endocarditis (IE). A 70-year-old man with chronic immune thrombocytopenia (ITP) presented to our emergency department with acute onset of altered mental status and right-sided weakness. He was afebrile, with no heart murmurs or peripheral IE stigmata. Neurologic examination showed disorientation, expressive aphasia, and right-sided hemiparesis. Laboratory findings were unremarkable except for leukocytosis and hyponatremia. Brain MRI showed an irregular rim-enhancing lesion in the left frontal lobe, suspicious for a high-grade glial neoplasm. The lesion was excised, and he was started empirically on vancomycin, ceftriaxone, and metronidazole. After blood cultures grew *Listeria monocytogenes*, antibiotics were de-escalated to ampicillin and gentamicin. Echocardiography showed mitral valve vegetation. By Day 6, his mental status had improved. On Day 9, he was discharged to our inpatient rehabilitation center to complete six weeks on IV ampicillin and IV gentamicin. Pathology of the brain mass was subsequently reported as a listerial brain abscess. Chronic treatment with high-dose oral glucocorticoids and pre-existing ITP have been independently implicated as predisposing factors in listerial brain abscess. There is a propensity to misdiagnose listerial brain abscess as an intracranial neoplasm due to similar clinical/imaging findings. In addition, *Listeria monocytogenes *is an atypical cause of IE. Therefore, a high index of suspicion is necessary for early recognition and successful treatment of listerial brain abscess and listerial endocarditis in high-risk patients.

## Introduction

*Listeria monocytogenes* is a facultatively anaerobic, non-spore-forming, gram-positive coccobacillus [1-3]. It is ubiquitous in nature with a broad host range. The bacterium has been isolated from soil, vegetation, and as part of the fecal flora in many mammals [1,3-6]. It is also normally found in the stools of 1-5% healthy adults [7,8]. It is pathogenic only in humans, with serotypes 1/2a, 1/2b, and 4b accounting for most infections [1,2].

*Listeria monocytogenes* is transmitted through consumption of contaminated foods including processed/delicatessen meats, soft cheeses, pâtés, vegetables, ice creams, unpasteurized milk, raw or ready-to-eat seafood, vegetables, and fruits such as cantaloupe [1,3,4]. It can grow over a wide variety of temperatures. It is more motile at lower temperatures between -2°C (28.4°F) and -42°C (-43.6°F), compared to a normal body temperature of 37°C (98.6°F) [1,3,9]. The bacterium can withstand harsh temperatures and low pH environments, thereby resisting killing by several food processing technologies [3,4,9]. It is weakly β-hemolytic on blood agar and is diagnosed through the culture of body fluids (specifically blood, cerebrospinal fluid, amniotic fluid) or brain tissue [1,10].

Recently, the incidence of listeriosis has been increasing. Disease severity depends on the age and immune status of the individual [2,9]. The spectrum of disease manifestations ranges from self-limiting gastroenteritis in young, healthy adults to life-threatening/fatal diseases including bacteremia, meningitis, encephalitis, and focal central nervous system (CNS) abscesses in high-risk groups such as neonates, pregnant women, and immunosuppressed patients [1,3]. Additional risk factors for severe disease include organ transplantation, cancer, chemotherapy, and treatment with steroids or tumor necrosis factor (TNF) alpha antagonists. However, up to 20% of cases of listeria brain abscess are reported to occur in patients without any identified underlying risk factors [5].

*Listeria monocytogenes* has a predilection for the CNS [10], with meningitis as the most common presentation especially in patients with altered cell-mediated immunity [10-12]. Brain abscess is a serious but rare manifestation of listerial infection, accounting for approximately 1-10% of cases [5,10]. From 1968 to 2017, only 73 cases of listerial brain abscess were reported in the literature, with a case fatality rate of 27.3% [5].

Furthermore, endocarditis due to *Listeria monocytogenes* is a rare entity occurring in only about 5-8% of patients with listeriosis [2,4,13]. A review of the literature from 1955 to 2008 found only 68 case reports of listerial endocarditis [14], with a case fatality rate ranging from 37-50% [4,6].

We present the case of an elderly man with both listerial brain abscess and endocarditis - a rare finding of two uncommon manifestations of listeriosis in the same patient.

## Case presentation

The patient is a 70-year-old Caucasian male resident of New York State with a history of diabetes mellitus, asthma, and chronic immune thrombocytopenia (ITP) that was refractory to medical management with high dose steroids and fostamatinib. He presented with a same-day history of acute mental status change and right-sided weakness associated with a shuffling gait. He had no history of recent travel, no recent tick or mosquito bites, no recent organ transplant, no preceding sinusitis or upper respiratory symptoms, and no head trauma. Of note, one week earlier, he had undergone right hemicolectomy for a cecal mass with high-grade dysplasia and splenectomy for ITP. He was discharged two days preceding this presentation, with histologic diagnosis of the colonic mass still pending.

**Figure 1:**
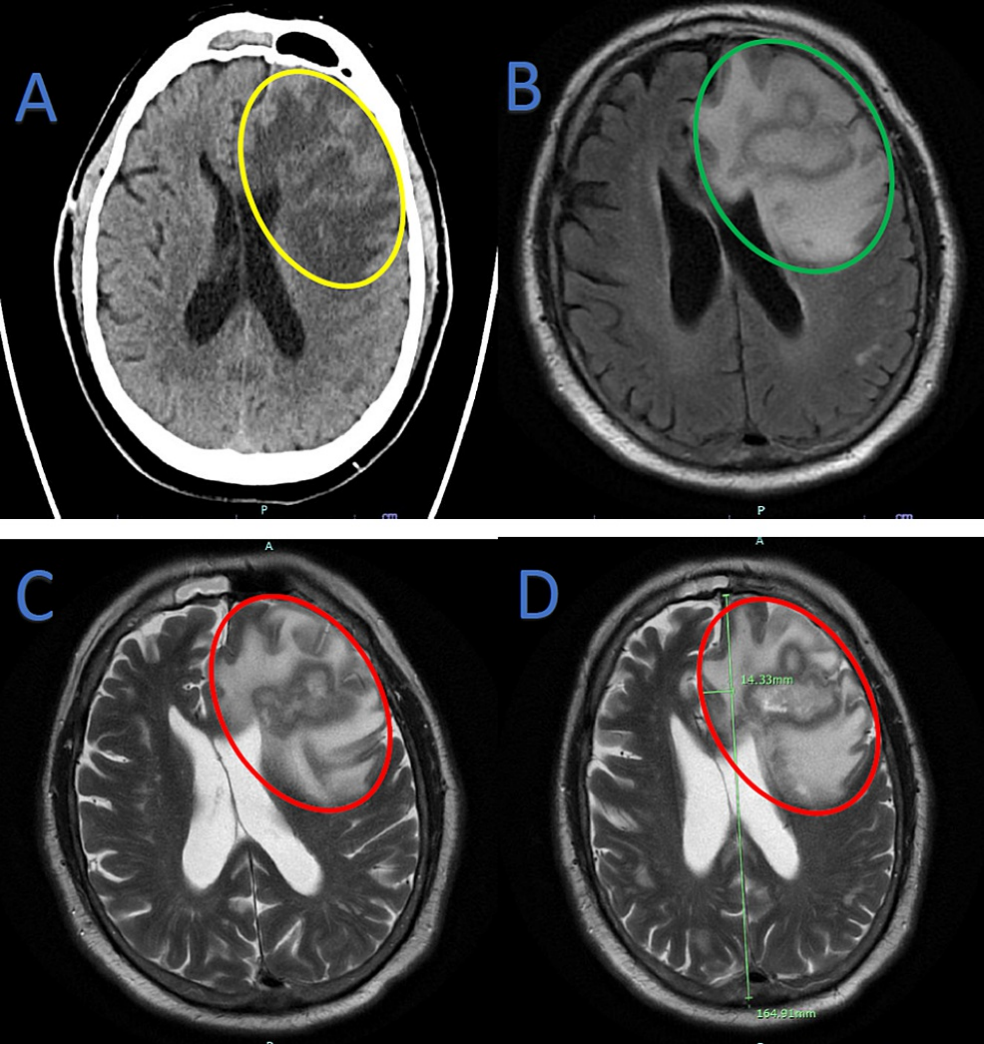
Brain imaging A: Non-contrast CT Head. Acute space-occupying mass effect in the left frontal lobe (yellow oval) with surrounding edema causing left-to-right midline shift, suspicious for malignancy. B-D: MRI Brain with and without contrast. An acute highly irregular rim-enhancing lesion in the left frontal lobe with surrounding edema and mass effect causing left-to-right midline shift, suspicious for high-grade infiltrative necrotic glioma. *Green oval:* without contrast; *Red ovals:* with contrast. *Green line* in D shows midline.

At presentation, the patient was afebrile (98.8 °F), and his other vitals were stable. Neurologic exam findings were remarkable for disorientation, expressive aphasia, reduced grip strength, and reduced power (4/5) in his right upper and lower extremities. Cardiovascular examination revealed no heart murmurs, and he had no clinical signs suggestive of septic emboli. Laboratory findings were notable for leukocytosis (13.7 x10^3^ cells/µL) and absolute neutrophilia (13.0 x10^3^ cells/µL), reactive post-splenectomy thrombocytosis (567 x10^3 ^cells/µL), hyperglycemia (244 mg/dL), hyponatremia (130 mmol/L), and hypoalbuminemia (3.1 g/dL). CT head without contrast showed an acute space-occupying mass effect in the left frontal lobe with surrounding edema causing left-to-right midline shift (Figure 1A). A subsequent MRI of the brain with and without contrast (Figure 1B-1D) showed an acute highly irregular rim-enhancing lesion in the left frontal lobe with surrounding edema and mass effect causing left to right midline shift. The patient was admitted and underwent an emergent craniotomy, with excision of a left frontal brain mass highly suspicious for a high-grade glial neoplasm. There was significant necrosis suggestive of a cerebral abscess. Hence, blood cultures were obtained, and specimens of the brain mass were sent for pathology.

**Figure 2:**
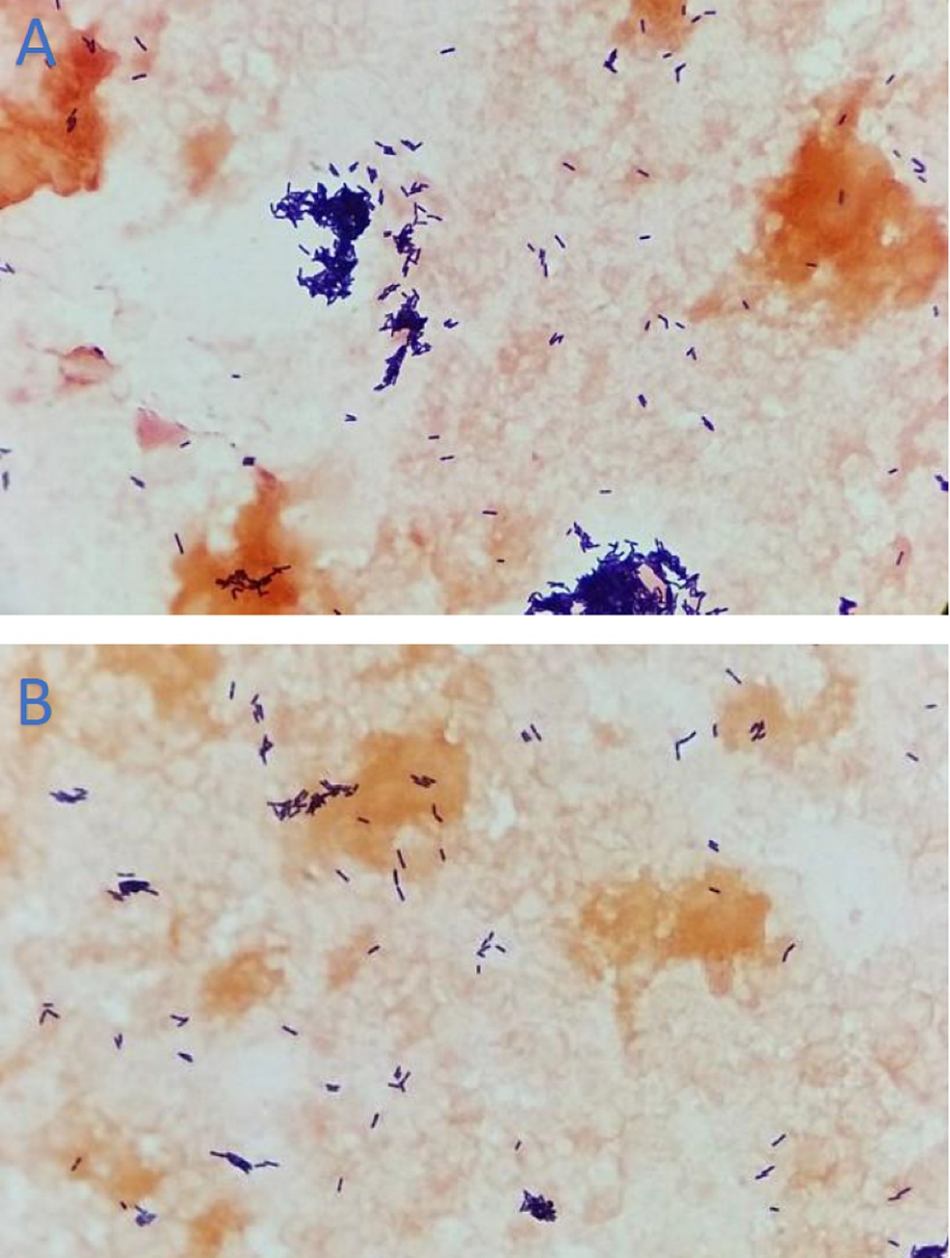
Gram stain of blood culture specimen showing Gram-positive rods A: View showing more clustered distribution of Gram-positive rods. B: View showing more disperse distribution of Gram-positive rods

On Day 2, blood cultures were notable for gram-positive rods (Figure 2), and he was started empirically on IV vancomycin, ceftriaxone, and metronidazole. Blood cultures subsequently revealed *Listeria monocytogenes* and antibiotics were de-escalated to IV ampicillin and gentamicin. Blood cultures that were drawn on Day 3 also grew *Listeria monocytogene*s. Following an unremarkable transthoracic echocardiogram (TTE), transesophageal echocardiography (TEE) showed a small mitral valve vegetation with no intracardiac abscess or significant mitral regurgitation. The patient developed fever on Day 4, but on Day 6, he began to defervesce, and his mental status showed improvement. On Day 9, he was transferred to the inpatient rehabilitation center of the hospital. There he completed a six-week course of IV ampicillin and gentamicin.

Pathology of the brain mass, which was received on Day 12, identified listerial brain abscess with no evidence of malignancy. On Day 14, the New York State Department of Health confirmed that real-time polymerase chain reaction (PCR) performed on the patient’s blood specimen was positive for *Listeria monocytogenes* DNA.

## Discussion

*Listeria monocytogenes* has a tropism for the brain parenchyma and the meninges. Besides meningitis, CNS manifestations include rhombencephalitis, granulomatous encephalitis, and macroscopic brain abscesses [15]. Listerial brain abscesses are uncommon. Involvement of subcortical gray matter, such as the basal ganglia and thalamus, is more commonly seen in listerial brain abscesses compared to other causes of brain abscess. Individuals with impaired cell-mediated immunity are at considerable risk of developing this disease. The predisposing factors that placed our patient at risk included his age, his chronic ITP, as well as his ITP treatment with high-dose prednisone and fostamatinib. Corticosteroids are associated with lymphopenia due to a reduction in the proliferative response of T-cells, leading to a decreased ratio of CD4+ cells to CD8+ cells peripherally. There is a significant correlation between the degree of lymphopenia and the daily steroid dose [16]. In a review of multiple case reports of listerial brain abscess, prior treatment with high-dose oral glucocorticoids was reported as a risk factor [5]. A prior diagnosis of ITP has also been independently identified as an underlying condition predisposing a patient to listerial brain abscess [5].

About 10% of focal CNS infections due to listeria are macroscopic abscesses that result from hematogenous spread, as seen in our patient [1,12]. In a review of 39 cases of listeria brain abscess, 38% of cases had concomitant meningitis and 86% had concomitant bacteremia [12]. Focal CNS abscesses due to *Listeria monocytogenes* often present with ataxia and hemiparesis [10]. The differential diagnosis often includes neoplasm, as seen in the case of our patient. Brain abscesses are usually associated with systemic symptoms such as fever and leukocytosis and have a more acute presentation compared to intracranial neoplasms. Nonetheless, systemic symptoms are reported in only about 50% of all patients with brain abscess, regardless of the causative agent. Hence, the diagnosis of brain abscess can be missed in patients who are afebrile or have other clinical features suggestive of neoplasm, such as weight loss, or unknown cancer lesion at another body site [1,5]. In our patient, who had recently undergone hemicolectomy for a cecal mass with pending pathologic diagnosis at the time of craniotomy, the suspicion of a primary or metastatic brain tumor was high. However, the resected cecal mass was eventually reported to be non-malignant.

The clinical presentation of focal CNS infections due to *Listeria monocytogenes* is usually biphasic - involving a prodromal phase characterized by fevers and headache followed by a neurologic phase characterized by altered mental status and focal neurologic findings [1]. Our patient did not have any of the symptoms associated with the prodromal phase. The radiological modality of choice for diagnosing brain abscess is MRI with gadolinium contrast [1]. Characteristically, ring-enhancing lesions are seen with listerial brain abscess, as observed in our patient [17]. Prompt diagnosis and prompt treatment of patients with listerial infection often result in full recovery [1]. But permanent neurologic complications are common in patients with brain abscess or rhombencephalitis.

A unique feature of this case is that, in addition to brain abscess, our patient also had evidence of endocarditis and satisfied two of the major Dukes criteria [18]. First, he was found to have listerial bacteremia from more than one blood culture specimen. Second, TEE showed vegetation on the mitral valve. Listerial endocarditis is usually associated with predisposing factors such as underlying rheumatic valve disease, prosthetic valves, or other structural heart diseases such as hypertrophic obstructive cardiomyopathy [11]. However, our patient had none of these predisposing heart conditions. The aortic valve and the mitral valve (i.e., the left-sided valves) are usually affected in listerial endocarditis, regardless of whether they are native or prosthetic valves [2,4,6].

The treatment modality of choice for brain abscess and/or infective endocarditis in listeriosis consists of a combination of ampicillin and gentamicin. The duration of treatment ranges from six to eight weeks [2]. Alternative effective antibiotic regimens, based on antimicrobial susceptibility profiles in patients with penicillin allergy or who have chronic kidney disease, include meropenem, daptomycin, or trimethoprim-sulfamethoxazole [2].

For unclear reasons, the incidence of both listerial brain abscesses and endocarditis is higher in men [2]. Our patient had no known prior history of consuming any types of food commonly implicated in listerial infection. The absence of information regarding the food source involved has also been reported in several sporadic cases of listerial infection that resulted in endocarditis or brain abscess [2]. This is possibly related to the highly variable incubation period of the organism (ranging from one to 70 days) and the many types of food that it is known to contaminate [3]. However, the median incubation period has been reported to be shorter in cases of bacteremia (median of two days, range of one to 12 days) and CNS infection (median of nine days, range of one to 14 days) [3].

## Conclusions

A high index of suspicion is necessary to identify and treat listerial brain abscess and listerial endocarditis in patients with risk factors for severe listeriosis manifestations. Even when patients do not fall within well-defined high-risk categories, it is still important to investigate appropriately and treat empirically when clinical, laboratory, and/or imaging findings suggest possible listeriosis. Although listerial brain abscess and listerial endocarditis are independent life-threatening conditions, morbidity and mortality can be minimized. Patients can have good outcomes if the appropriate antibiotic regimen is started promptly and continued for the recommended duration.
